# Gender associated differences in determinants of quality of life in patients with COPD: a case series study

**DOI:** 10.1186/1477-7525-4-72

**Published:** 2006-09-28

**Authors:** Juan P de Torres, Ciro Casanova, Concepción Hernández, Juan Abreu, Angela Montejo de Garcini, Armando Aguirre-Jaime, Bartolome R Celli

**Affiliations:** 1 Respiratory Research Unit, Hospital Nuestra Sra de Candelaria, Tenerife, Spain; 2 Pulmonary and Critical Care Division, St. Elizabeth's Medical Center, Boston, USA

## Abstract

**Background:**

The influence of gender on the expression of COPD has received limited attention. Quality of Life (QoL) has become an important outcome in COPD patients. The aim of our study was to explore factors contributing to gender differences in Quality of Life of COPD patients.

**Methods:**

In 146 men and women with COPD from a pulmonary clinic we measured: Saint George's Respiratory Questionnaire (SGRQ), age, smoking history, PaO_2_, PaCO_2_, FEV_1_, FVC, IC/TLC, FRC, body mass index (BMI), 6 minute walk distance (6MWD), dyspnea (modified MRC), degree of comorbidity (Charlson index) and exacerbations in the previous year. We explored differences between genders using Mann-Whitney U-rank test. To investigate the main determinants of QoL, a multiple lineal regression analysis was performed using backward Wald's criteria, with those variables that significantly correlated with SGRQ total scores.

**Results:**

Compared with men, women had worse scores in all domains of the SGRQ (total 38 vs 26, p = 0.01, symptoms 48 vs 39, p = 0.03, activity 53 vs 37, p = 0.02, impact 28 vs 15, p = 0.01). SGRQ total scores correlated in men with: FEV_1_% (-0.378, p < 0.001), IC/TLC (-0.368, p = 0.002), PaO_2 _(-0.379, p = 0.001), PaCO_2 _(0.256, p = 0.05), 6MWD (-0.327, p = 0.005), exacerbations (0.366, p = 0.001), Charlson index (0.380, p = 0.001) and MMRC (0.654, p < 0.001). In women, the scores correlated only with FEV_1_% (-0.293, p = 0.013) PaO_2 _(-0.315, p = 0.007), exacerbations (0.290, p = 0.013) and MMRC (0.628, p < 0.001). Regression analysis (B, 95% CI) showed that exercise capacity (0.05, 0.02 to 0.09), dyspnea (17.6, 13.4 to 21.8), IC/TLC (-51.1, -98.9 to -3.2) and comorbidity (1.7, 0.84 to 2.53) for men and dyspnea (9.7, 7.3 to 12.4) and oxygenation (-0.3, -0.6 to -0.01) for women manifested the highest independent associations with SGRQ scores.

**Conclusion:**

In moderate to severe COPD patients attending a pulmonary clinic, there are gender differences in health status scores. In turn, the clinical and physiological variables independently associated with those scores differed in men and women. Attention should be paid to the determinants of QoL scores in women with COPD.

## Background

Chronic Obstructive Pulmonary Disease (COPD) primarily affects the airway and lung parenchyma while it also induces clinically important systemic consequences. For an appropriate diagnosis and follow up a multidimensional evaluation of the disease is required including: degree of airway obstruction, lung hyperinflation, dyspnea, exercise capacity, quality of life and nutritional status.

The influence of gender on the clinical expression of COPD has received limited attention. The lack of information regarding gender and COPD is surprising, because according to the recent COPD disease surveillance in the United States [1], for the first time in 2002, the number of women dying from this disease surpassed that of men.

Quality of Life has become an important measurable outcome in patients with Chronic Obstructive Pulmonary Disease (COPD). It is known to predict mortality [2], hospitalization [3], health care resource utilization [3] and response to different treatment options [4]. The Saint George's Respiratory Questionnaire (SGRQ) has become one of the most widely used health-related specific questionnaires for assessing QoL in respiratory patients [5].

In the literature there are few reports suggesting a greater impairment in health related quality of life in female patients with COPD [6-10]. Several studies completed mainly in men with COPD, have explored the physiological and psychological factors associated with QoL impairment [11-14]. They have shown that dyspnea, six minute walk distance (6MWD), PaO_2_, FEV_1_, anxiety and depression are associated with the QoL scores in these patients.

In a previous study [15], we found that in a FEV_1_% matched population of COPD patients, women had worse SGRQ scores than men at younger age and earlier stage of the disease. We planned the present study in a larger sample, to explore possible gender differences in the factors associated and predictive of SGRQ scores in both genders.

## Methods

This FEV_1 _matched case series study, recruited COPD patients attending the pulmonary clinic at Hospital Universitario Ntra Sra de Candelaria, a tertiary public university hospital in Spain from January 2000 to December 2005. We recruited 73 consecutive women attending the clinic and then matched 73 patients with similar degree of airflow obstruction randomly selected from our much larger population of men with COPD. Patients with all degree of airflow severity were included if they had smoked ≥ 20 pack years and had a post-bronchodilator FEV_1_/FVC of <0.7 after 400 micrograms of inhaled albuterol. Patients were excluded if they had a history of asthma, has a history of bronchiectasis, tuberculosis or other confounding diseases. The patients were clinically stable (no exacerbation for at least 2 months) at the time of the evaluation and were part of the population studied for the BODE international multicenter study [16]. The Ethical Committee of the Hospital approved the study and all patients signed the informed consent.

We evaluated the QoL of the study sample by the SGRQ. We also measured proven prognostic parameters for COPD patients: age, degree of airflow obstruction by FEV_1_, dyspnea by the Modified Medical Research Council scale (MMRC), exercise capacity by the Six Minute Walk Distance (6MWD) and the presence of comorbidities by the combined Charlson index [17] where the higher the score, the more co-morbidities are present, and the exacerbations in the previous year of the study date.

Postbronchodilator FEV_1_, FVC, FEV_1_/FVC and IC/TLC were determined taking the European Community for Steel and Coal for Spain reference [16] using a Jaegger 920 MasterLab^® ^Body Box. BMI was calculated as the weight in kilograms divided by height in meters^2^. Arterial blood gases were measured at rest.

Exacerbations were defined as episodes of increased dysnea, production of phlegm and cough that required medical attention, differentiating those that required admission and those that did not for one full year.

The 6MWD was performed following the ATS guidelines [19] using as reference values those of Troosters et al [18]. Functional dyspnea was measured using the ATS modified MMRC [21]. Health status was determined using the language-specific validated SGRQ questionnaire that provides three individual domain scores: symptoms, activity, impact (psychosocial dysfunction). A total score is calculated, with zero indicating no impairment and 100 representing maximum impairment [5].

We used the following gender matching method: from an initial sample of 110 males and 73 females with COPD; we were able to match every female patient with a male with FEV_1_% of predicted ± 2%; when more than one male matched, we chose the male patient to be included in the final sample by random drawing from a list while being blinded to the rest of the parameters included in this study. The matching process was done prospectively and at the time of diagnosis. A sample of 73 patients in each group allowed us to detect a relevant difference as small as 10 points for SGRQ scores, in a two-tailed test at 5% significant level with a power of 85%, considering a median SGRQ value of 30 points for men and 40 for women.

We describe each variable using mean ± SD or median (25^th ^percentile-75^th ^percentile) depending on their distribution. We explored for differences between genders in each parameter using Student t-test for variables with approximately normal distribution, Mann-Whitney U-rank test for variables without normal distribution. We then correlate SGRQ Total scores with the different studied variables. A multiple linear regression analysis was performed using backward Wald's criteria, with those variables that significantly correlated with SGRQ Total scores. A p value ≤ 0.05 was considered statistically significant.

## Results

The patients were white Caucasian and when enrolled, 25% of the men and 23% of the women were still smoking. None of the patients had a history of exposure to biomass fuel. Using the GOLD staging system [22] we have equal number of men and women in each GOLD Stage (I 13%, II 43%, III 36%, IV 5%).

The clinical and physiological characteristics of the patients participating in the study are described in Table 1. Women were younger and smoked less than men. There were no differences in current smoking status. Women had lower BMI and a higher percentage of them had a BMI ≤ 21. Women had less co-morbidities and more exacerbations in the previous year than men. No differences were found in FRC% predicted. Women had a higher PaO_2 _and lower PaCO_2 _than men. Even though they had the same predicted FEV_1 _and better mean PaO_2_, women had a lower 6MWD in % of predicted values and reported more functional dyspnea. They also scored worse in all domains of the SGRQ.

**Table 1:** Comparisons of clinical and physiological characteristics between women and men matched by their predicted FEV_1_

**Clinical & Physiological Characteristics**	**Men (n = 73)**	**Women (n = 73)**	**p Value**
Age (years old)	63 ± 8*	56 ± 11*	<0.001
Age range	47–77	37–79	
Pack-years history	69 ± 26*	47 ± 22*	<0.001
BMI (kg/m^2^)	27 ± 4*	25 ± 7*	0.04
BMI ≤ 21(%)	6	32	0.007
Charlson Index (points)	3 (1–6) +	1 (1–3) +	<0.001
MMRC (points)			
0–2 (%)	93	71	<0.001
3–4 (%)	7	29	
Exacerbations in the last year			
Without admission	0 (0–1) +	1 (0–2) +	0.013
With admission	0 (0-0) +	0 (0–1) +	0.116
FRC % of predicted	142 ± 31*	134 ± 32*	0.470
IC/TLC ≤ 0.25 (%)	8 (11)	7 (10)	0.627
PaO_2 _(mmHg)	70 ± 11*	76 ± 11*	0.004
PaCO_2 _(mmHg)	45 ± 6*	40 ± 5*	<0.001
6MWD (mts)	529 ± 93*	459 ± 79*	<0.001
6MWD % of Predictive	107 ± 21*	85 ± 17*	<0.05
SGRQ			
Total	26 (15–52) +	38 (30–47) +	0.011
Symptoms	39 (12–53) +	48 (32–58) +	0.028
Activity	37 (14–62) +	53 (40–62) +	0.020
Impact	15 (6–45) +	28 (13–40) +	0.013

*represents mean ± SD; + represents median and 25^th^-75^th ^percentiles

When we compared SGRQ scores for the population divided by FEV_1_% in greater and lower than 50%, there were gender differences only for the group with FEV_1 _>50% group [n = 43 for each gender, p < 0.05 in all comparisons, men and women respectively: total 17(6–30) vs 32 (25–42), symptoms 31(11–44) vs 42(28–56), activity 23 (8–40) vs 53 (43–56) and impact 8 (5–26) vs 20 (13–35)]. We did not find differences for the more severe group of patients (FEV1% <50%, GOLD stages III and IV).

Those parameters that significantly correlated with SGRQ total scores are shown in Table 2 for the entire population and divided by gender. There were gender differences in the parameters that correlated with SGRQ.

**Table 2:** Studied parameters that significantly correlated with SGRQ total scores.

**Studied parameters**	**Entire population**	**Male**	**Females**
FEV_1_% of predictive	-0.378 (p < 0.001)	-0.479 (p < 0.001)	-0.293 (p = 0.013)
IC/TLC	-0.306 (p = 0.001)	-0.368 (p = 0.002)	NS
PaO_2 _(mmHg)	-0.269 (p = 0.001)	-0.379 (p = 0.001)	-0.315 (p = 0.007)
PaCO_2 _(mmHg)	NS	0.256 (p = 0.057)	NS
6MWD (mts)	0.267 (0.002)	-0.327 (p = 0.005)	NS
Exacerbations	0.343 (p < 0.001)	0.366 (p = 0.001)	0.290 (p = 0.013)
Charlson Index	0.210 (p = 0.012)	0.380 (p = 0.001)	NS
MMRC (points)	0.659 (p < 0.001)	0.654 (p < 0.001)	0.628 (p < 0.001)

We show correlation coefficients and p values for those that showed statistical significant correlation

Spearman's coefficients for all correlations

**Figure 1:**
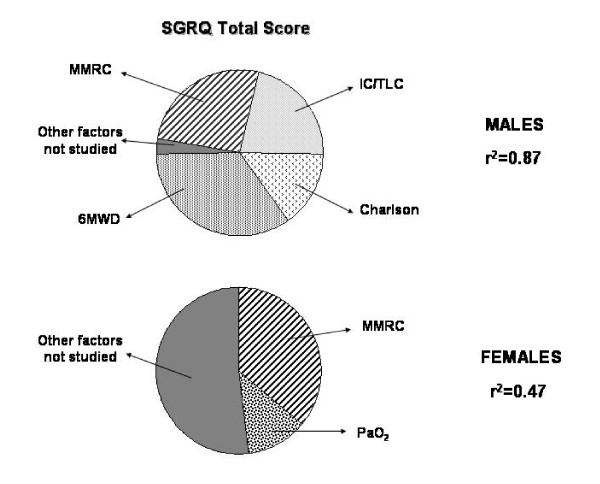
The diagrams shows the relative weight of the factors retained in the logistic regression analysis as predictors of the SGRQ total scores for male and female COPD patients. SGRQ total = Saint George's Respiratory Questionaire total score. MMRC = Modified Medical Research Council scale. 6MWD = Six minute walking distance test. Charlson = Charlson index. IC/TLC = Inspiratory Capacity/Total Lung Capacity ratio. PaO_2 _= Arterial oxygen pressure.

Table 3 shows multiple linear regressions of those factors that significantly correlated with SGRQ Total scores divided by gender. Once again, the factors that predict SGRQ total scores differed by gender. Figure 1 shows the relative weight of the studied factors retained in the multiple linear logistic regression analysis as predictors of the SGRQ total scores for male and female COPD patients. We used the β coefficients of the parameters retained in the regression model to calculate de proportional weight that each has to predict the variance of the SGRQ total score.

**Table 3:** Multiple lineal regressions with parameters that significantly correlated with SGRQ total

**Quality of Life**	**Group**	**Parameter**	**B (95%CI)**	**p value**
SGRQ total	Entire population r^2 ^= 0.52	Charlson	1.63 (0.89–2.37)	<0.001
		MMRC	14.6 (11.7, 17.4)	<0.001
	Males r^2 ^= 0.87	Charlson	1.68 (0.84, 2.53)	<0.001
		IC/TLC	-51.1 (-98.9, -3.2)	0.037
		MMRC	17.6 (13.4–21.8)	<0.001
		6MWD	0.05 (0.02–0.09)	0.002
	Females r^2 ^= 0.48	MMRC	9.7 (7.3–12.4)	<0.001
		PaO_2_	-0.3 (-0.6, -0.01)	0.042

## Discussion

The most important finding of this study is that in moderate to severe COPD patients attending a pulmonary clinic the factors associated with SGRQ total score are different in men and women. Whereas dyspnea, exercise capacity, degree of hyperinflation and comorbidity show an independent association with the scores in men, only dyspnea and level of arterial oxygenation contributed to the score in women.

The information about gender differences in QoL of patients with COPD is scarce [6-10]. Osman et al included 266 severe COPD (123 men, 115 women) to investigate if QoL (measured by the SGRQ) could predict hospital readmission. Even though this was not an FEV_1 _matched population and comparison of gender differences was not the main goal of the study, they noted worse scores in women than in men. Leidy et al compared the functional performance of 45 women and 44 men with COPD using the Sickness Impact Profile. They reported no significant differences in all categories but indicated gender differences in models of functional performance. Larson and co-workers also reported worse QoL scores in women. Rodrigue et al [9] showed that in a population of COPD who underwent lung transplantation, women reported worse scores and less improvement in QoL after surgery although they had a greater improvement in their spirometric values. Recently Di Marco et al [10] reported in a population of 202 COPD patients, worse symptom-related QoL, and more anxiety and depressive symptoms in female patients compared with men. However, all of these authors did not match for degree of airflow limitation and they did not explore differences in the factors that could help explain the worse scores in women.

As an extension to our previous published study [15], we planned this study to investigate the possible factors associated to this gender differences. We observed that SGRQ scores in all domains were higher in female patients than men. The gender differences were all higher than the 4 points considered clinically significant for SGRQ [23]. Surprisingly, when we classified the patients by severity of obstruction into FEV_1_% greater and lower than 50%, only women with mild to moderate disease (GOLD stages II and II) had higher (worse) scores in all domains of the SGRQ, than the men. There were no differences in any domain for Stage III and IV patients.

This is an interesting finding considering that female with FEV_1_% <50% were younger than males (53 ± 9 vs. 66 ± 8, p < .001). We interpreted this observation as indicating that women with COPD develop symptoms influencing the SGRQ questionnaire at a younger age and with less degree of obstruction than men. Classically, we know that QoL impairment starts to be noticed when FEV_1_% falls below 50% [24]. Our findings imply that females with COPD differ from males in having an earlier repercussion of the disease (even at predicted FEV_1 _values between 65–80%). This suggests that we should pay more attention to the early detection of the disease in women. Indeed, the early age of onset of impairment in QoL in women should raise alarm considering that most of the primary care physicians do not think in COPD when they see females with typical symptoms of cough, phlegm or dyspnea [25]. It is also important since the impairment of QoL in female could run for longer time and the response to different treatment options aimed at improving QoL, like pulmonary rehabilitation, are not the same in females and males with COPD [26].

In this study we also show that the variables that correlated with SGRQ scores differed by gender (Table 2). If we only consider the SGRQ total score as a summary of the QoL expression, the parameters that correlated in men (FEV_1_, IC/TLC, PaO_2_, PaCO_2_, exacerbations, Charlson, 6MWD and MMRC) were different from those in women (FEV_1_, PaO_2_, exacerbations and MMRC). Our results are in-line with those reported by Tsukino et al [9] in a predominatly male COPD cohort, which provides external validity to our findings. We then can speculate that the factors affecting QoL differ by gender at least in the early stages of the disease and that the perceived expression of the disease is different between genders.

Table 3 summarizes the associated predictors of SGRQ total scores for males and females with COPD with the same degree of airway obstruction. The difference between genders constitutes the most important finding of our work. While factors like dyspnea, exercise capacity, degree of hiperinflation and comorbidities explain almost 90% of the variation of the SGRQ total score in our male patients, dyspnea and level of arterial oxygenation only explained 50% of the variation of it in the female population. It suggests that the female COPD population is entirely different and that we should look for possible factors to be included in their regular evaluation to try to explain the greater and earlier impairment of their QoL.

Dyspnea continues to be the most important driving force of the QoL impairment in patients with COPD and therapies aiming at relieving this cardinal symptom are important in COPD women as well as the close follow up of their degree of arterial oxygenation. We know that psychological factors have an important impact in QoL of COPD patients [13], with a higher prevalence of depression and anxiety in female COPD patients [10]. We also know from previous works that female coping mechanisms with COPD are different that those from males [7]. We then especulate, as also recently suggested by Di Marco et al [10] that the evaluation of factors like the psychological or socio-cultural ones are possible venues that should be investigated in the female COPD population in order to explain their impaired QoL.

We believe our study has several limitations. First, our patients were recruited from those attending a pulmonary clinic and therefore may not represent the COPD population at large. Second, our findings in women may only be applicable to patients with cigarette related COPD and not to patients with COPD due to biomass fuel [27]. Third, we did not include depression and anxiety evaluations in the parameters considered, because the study was designed to explored physiologic factors previously associated with health-related quality of life in patients with COPD. Also, we also did not include generic questionnaires like the SF-36, in the evaluation of the QoL of these patients, as some investigators believe are complementary of the specific ones. Considering the scarce information in the area of QoL in women with COPD, it would have been important to include them to better reflect all aspects of the QoL impairment. Lastly, our population study mainly represents GOLD stages II and III and conclusions can only be referred to this degree of obstruction. Nevertheless, the main differences found in SGRQ scores are in the early stages of the disease, and we believe the conclusions here found represent an important message because most of the patients seen at pulmonary clinics have similar characteristics as ours.

## Conclusion

In summary, our study shows that factors associated with QoL of moderate to severe COPD patients differ by gender. The main predictors of SGRQ total score in men are dyspnea, exercise capacity, degree of hyperinflation and comorbidity, whereas for women, the main predictors are dyspnea and level of arterial oxygenation. Most importantly, our data suggests that to appropiately evaluate QoL in women with COPD, prognostic factors other than the traditional ones should be included because these do not fully predict the health related quality of life scores.

## Competing interests

The author(s) declare that they have no competing interests.

## Authors' contributions

JdT conceived of the study, and participated in its design and coordination and helped to draft the manuscript. CC participated in the study design and coordination and helped to draft the manuscript. CH participated in the study design and coordination and helped to draft the manuscript. JA participated in the study design and coordination and helped to draft the manuscript. AM participated performing lung function test and the 6MWD. AAJ helped in the design of the study and the statistical analysis of the data. BC helped in the interpretation of the data and to draft the manuscript.
